# Temperature and Size Effect on the Electrical Properties of Monolayer Graphene based Interconnects for Next Generation MQCA based Nanoelectronics

**DOI:** 10.1038/s41598-020-63360-6

**Published:** 2020-04-10

**Authors:** Sanghamitra Debroy, Santhosh Sivasubramani, Gayatri Vaidya, Swati Ghosh Acharyya, Amit Acharyya

**Affiliations:** 1 Department of Electrical Engineering, Indian Institute of Technology, Hyderabad, India; 2 Department of Electronic Materials Engineering, Research School of Physics, The Australian National University, ACT, 2601 Australia; 3 School of Engineering Sciences and Technology, University of Hyderabad, Hyderabad, India

**Keywords:** Electronic devices, Electronic properties and devices, Electronic devices, Graphene, Electronic properties and devices

## Abstract

Graphene interconnects have been projected to out-perform Copper interconnects in the next generation Magnetic Quantum-dot Cellular Automata (MQCA) based nano-electronic applications. In this paper a simple two-step lithography process for patterning CVD monolayer graphene on SiO_2_/Si substrate has been used that resulted in the current density of one order higher magnitude as compared to the state-of-the-art graphene-based interconnects. Electrical performances of the fabricated graphene interconnects were evaluated, and the impact of temperature and size on the current density and reliability was investigated. The maximum current density of 1.18 ×10^8^ A/cm^2^ was observed for 0.3 μm graphene interconnect on SiO_2_/Si substrate, which is about two orders and one order higher than that of conventionally used copper interconnects and CVD grown graphene respectively, thus demonstrating huge potential in outperforming copper wires for on-chip clocking. The drop in current at 473 K as compared to room temperature was found to be nearly 30%, indicating a positive temperature coefficient of resistivity (TCR). TCR for all cases were studied and it was found that with decrease in width, the sensitivity of temperature also reduces. The effect of resistivity on the breakdown current density was analysed on the experimental data using Matlab and found to follow the power-law equations. The breakdown current density was found to have a reciprocal relationship to graphene interconnect resistivity suggesting Joule heating as the likely mechanism of breakdown.

## Introduction

Interconnects are going to play an important role in the next generation Magnetic Quantum-dot Cellular automata (MQCA) based nano-electronics^1–3^. Copper being the state-of-art interconnect material is facing severe challenges while being scaled down to nano dimensions due to its increased resistivity that is mainly because of its surface and grain boundary scatterings^4^ and also susceptibility to electromigration effect^5^. Moreover, copper as an on-chip clocking material requires high currents and large dimensions^6^ in order to generate the external field in MQCA. To illustrate, copper interconnects (CI) used in MQCA occupies more than 2000 nm in order to generate the required external field for data propagation between the nano-magnets^7^. Therefore CI impose limitations on the emerging nanoelectronic applications leading to extensive research in finding an alternate material that can replace the CI.

Thus in this context, graphene^8–14^ can be envisaged as a potential interconnect which could replace copper. Moreover, graphene is projected to be an excellent candidate material for interconnects due to its high carrier mobility ($2\times {10}^{5}$ cm^2^/V-s)^15^, ballistic transport^16^, high current carrying capacity^17^ and high thermal conductivity^18^. On the other hand, the length of the nanomagnets used in typical MQCA devices are more than 100 nm and therefore the underline interconnects should be in size ranging from more than 100 nm and less than the CI which are currently used.

CVD based multilayer and monolayer graphene interconnects have been investigated in^19,20^ resulting in the maximum current density of 4 ×10^7^A/cm^2^ and 1.2×10^7^ A/cm^2^ on SiO_2_ substrate respectively. This is an order of magnitude less than the state-of-the-art Graphene Nanoribbon where the size is typically much less than 100 nm hence, it is not suitable as interconnects in MQCA^17,21,22^. Thus in an attempt to increase the current density in the CVD grown graphene, several hybrid structures have been reported^23–27^ with an additional overhead of significant increase in the fabrication complexity. To address these issues we introduce here a simple fabrication procedure of patterning graphene interconnects in the context of next-generation MQCA, still retaining an order of magnitude higher current density as compared to the present CVD graphene interconnects.

Furthermore, in order to analyse the problem of integrating graphene interconnects in next-generation MQCA based nano-electronics, it is necessary to understand the effect of temperature and size impact on the electrical parameters. Graphene being projected as a potential alternative for interconnect material, the assessment of temperature and size would be of utmost importance. However, published literature lacks data on symbiotic effect of temperature and size on the electrical conductivity of graphene. Hence an organised attempt has been made here to understand the combined effect of temperature and size on the electrical behaviour of CVD grown monolayer graphene on SiO_2_/Si substrate. Thus in this paper we report-

1. a simple two-step lithography process for patterning CVD monolayer graphene interconnects resulting in one order of magnitude higher current density and
2. effect of temperature and size on the breakdown current density of CVD-monolayer graphene interconnects.

The rest of the paper is organised as follows: Section 2 describes the experimental setup, section 3 describes the results followed by its discussions and the section 4 draws the conclusion.

## Experimental details

State of the art, patterning of CVD based graphene interconnects involves a minimum of five lithographic processes i.e. two times optical and three times electron beam lithography^20^. This results in the usage of different polymer resist that leaves behind the resist residues in each step of the lithographic process thus reducing the current carrying capacity of the graphene interconnects.

**Figure 1:**
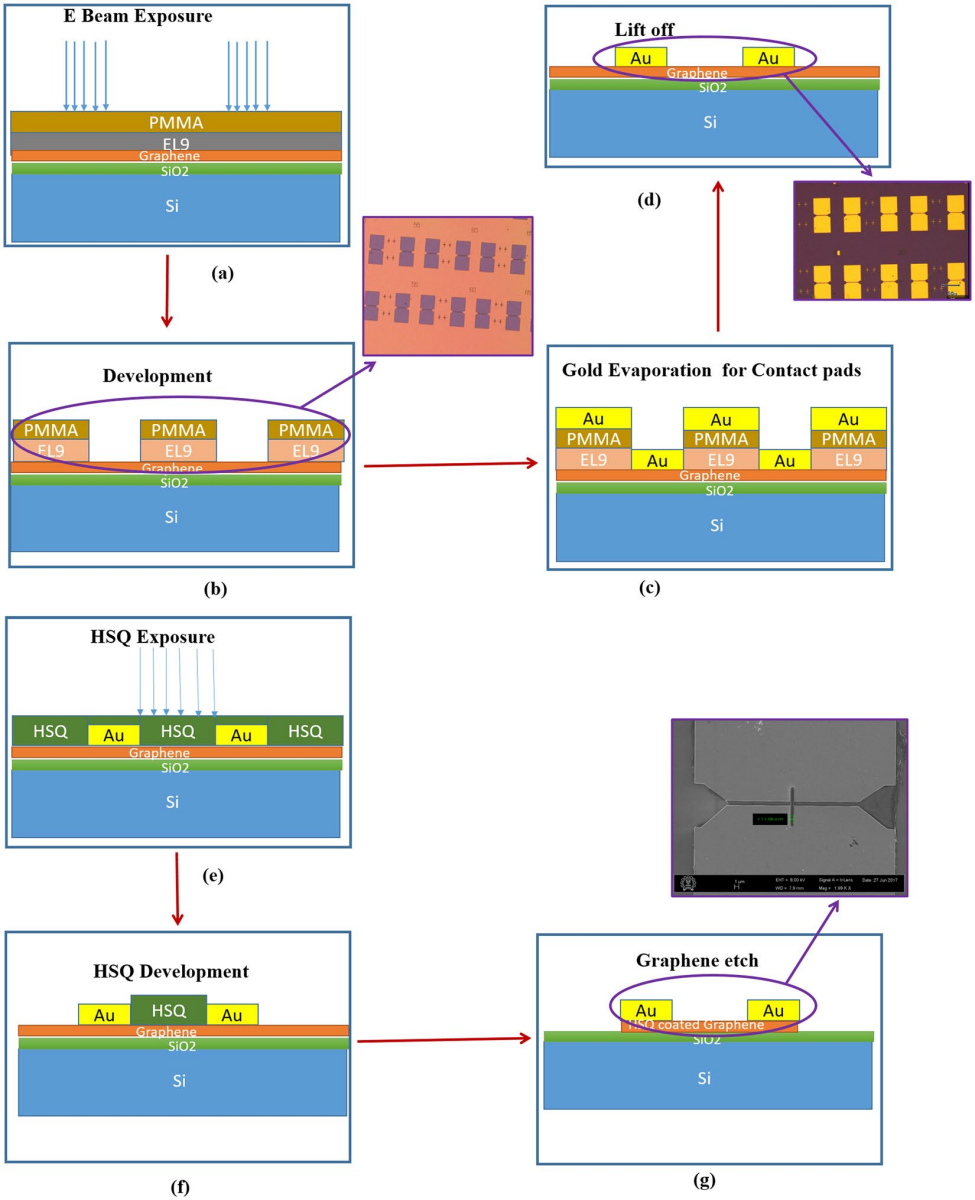
Fabrication process flow for patterning CVD graphene using a two-step approach - (**a–d**) shows the different stages of patterning the alignment marks and the contact pads and the insets shows the optical images of the same. (**e–g**) shows the patterning of the graphene interconnects and inset shows the SEM images of the patterned interconnect.

In this paper, we report a novel two-step lithography process for CVD monolayer graphene patterning. A detailed description with pictorial representation has been given in Fig. 1 so that it can be reproduced in future fabrication work with less experimental rigour compared to the state-of-the-art techniques.

Initially, we started with graphene monolayer (10 mm × 10 mm) produced by CVD on copper catalyst and transferred to a SiO_2_ (300 nm thickness)/Si substrate using wet transfer process that was procured from M/s. Graphenea Inc. USA. The grain size was approximately 10 μm for monolayer graphene with greater than 95% coverage and with small multilayer islands.

The first lithography step involved patterning the metal contacts and the alignments marks. The alignment marks and the metal contacts Cr/Au (10 nm/70 nm) were fabricated by electron-beam lithography (EBL). Bilayer EBL resist, has been used in the reported experiment in order to achieve better liftoff of Gold (Au). PMMA - a high-resolution e-beam resist and Copolymer EL9 is selected as the bilayer resist material for the EBL. The sample is exposed to an electron beam of acceleration voltage 16 kV and a dose of 180 μC/cm^2^. Figure 1(a) shows the detailed structure of the Bi-layer resist used in this experiment for better lift-off. EL9 was spun at 3200 rpm for 60 sec and Pre-Baked at 180 °C for 7 minutes followed by PMMA 950 K (4%) at 4000 rpm for 45 sec and bake it at 180 °C for 2 mins. The exposed resist was then developed in the MIBK (methyl isobutyl ketone) and IPA (Isopropyl alcohol) developer (1:3) for 30 s at room temperature followed by N_2_ drying. Subsequently metal deposition was carried out at a pressure of 10^−6^ Torr and then lift off was done in acetone for 4 hours.

The second lithography step involves patterning the graphene interconnects with width (W) in the range of 300 nm to 1500 nm, length of 1 μm and thickness of 0.335 nm. HSQ (Hydrogen Silsesquioxane) was used for patterning the graphene channels at 2000 rpm for 45 sec and exposing it to acceleration voltage of 18 kV and a dose of 190 s μC/Cm^2^. The resist was developed in Microposit MIF319 developer and Tetra-methyl ammonium hydroxide (TMAH 2.3%) for 1 sec. While using HSQ an overlying dielectric layer is formed on the graphene interconnects. A low power oxygen plasma etch for 1 min 30 sec and at 50 Watt power was used, but still the fine line remained. After the plasma etch the HSQ resist pattern was etched into the graphene flake.

The Current-voltage (I-V) characteristics of the device were measured using Proxima (Fast IV Measurement/B1500) and Keithley 4200 Semiconductor Characterization System with two-probe configuration. The (I-V) characterisation was done by sweeping voltage in steps of 0–0.5 V, 0–1 V followed by 0 to breakdown voltage. Due to increasing current density in the graphene interconnects, there was a voltage at which the break down occurred in the graphene interconnect, resulting in a visible drop in current. The device testing was stopped at this point. This way of measurement helps in suppressing the effect of trapping centres due to resist residuals. Breakdown voltage for all cases was nearly found at 2 V. The temperature measurements were carried out at 298–473 K respectively. Temperature vs I-V characterisation results are discussed in the following section.

However, it has been reported in^22^ that HSQ coating does not degrade the carrier mobility or rather they sometimes improve the mobility. Thus the reported two-step process of fabricating graphene interconnect not only reduces the rigour of fabrication but also reduces the residual trapping leading to higher current capacities of the graphene interconnects that is discussed in the subsequent section.

## Results and Discussion

SEM and Raman spectrum studies were conducted at several locations in order to confirm the uniformity of the monolayer graphene film.

**Figure 2:**
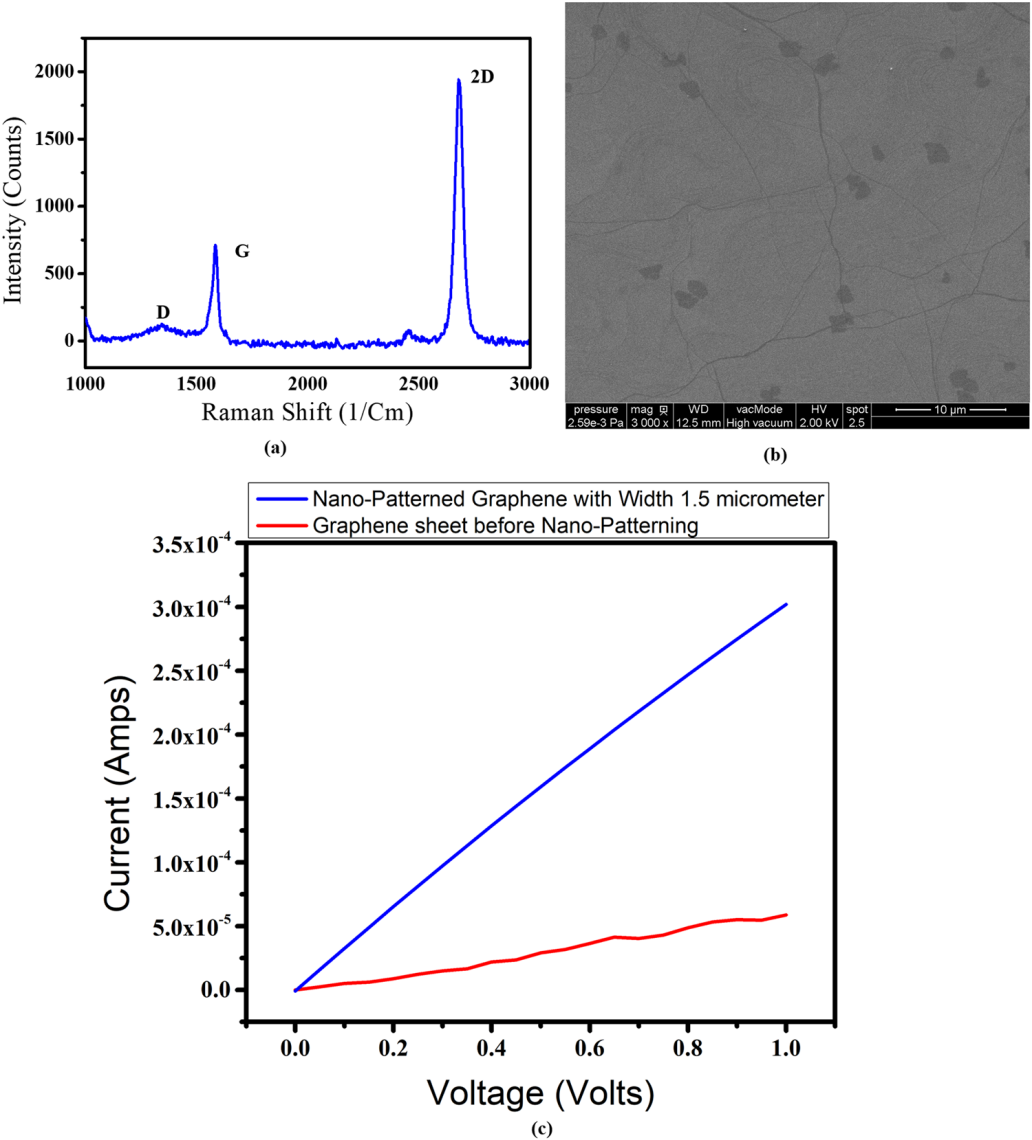
(**a**) Raman spectrum of the monolayer graphene sample (**b**) Scanning electron microscopy (SEM) image of graphene (**c**) Shows the I–V plot of the graphene sheet and graphene interconnect of width 1.5 μm and length 1 μm.

Figure 2(a) shows the Raman spectrum of the sample with an excitation wavelength of 532 nm (green, Ar laser), calibrated using quartz. The signature peaks, namely D, G and 2D bands, appeared around 1340, 1584 and 2800 cm^−1^, respectively. This ensures I(_G_)/I(_2D_) = 0.55, which indicates that it is a monolayer graphene sample^28–33^. Figure 2(b) gives the Scanning electron microscopy (SEM) image of graphene on top of SiO_2_ (300 nm/Si substrate).

In spite of graphene devices that are produced from highly oriented pyrolytic graphite (HOPG), laser ablation, spin coating, CVD graphene is widely known method for device fabrication since it is by far the most popular way for producing graphene and also results in relatively high quality graphene, potentially on a large scale^34–38^. Therefore we have used CVD Monolayer graphene film on SiO2/Si substrate procured from M/s. Graphenea Inc, USA, with a current density of 10^2^ A/cm^2^, having width of 1 Cm × 1 Cm × 0.35 nm as the starting material for patterning graphene interconnects.

Besides, study reported in^19,20^ have also used CVD graphene for electrical characterization of the patterned samples and the maximum current densities reported are up to 4×10^7^ A/cm^2^ and 1.2×10^7^A/cm^2^ respectively which is about an order lesser than that of our fabricated graphene interconnects with the maximum current density of 1.18 ×10^8^ A/cm^2^, which is attributed by the proposed simplistic two-step procedure.

The major bottleneck for graphene application is, while patterning due to multistep process - the electronic transport properties gets degraded due to the resist residual trapped in the grain boundaries and on the surface of the CVD graphene that acts as scattering sites, limiting the transport of charge carriers resulting in the reduction of the electron mobility and also increase in the sheet resistance of the CVD graphene sheet^20^.

Thus in this paper, a simple two-step lithography process has been reported for patterning graphene. As the number of lithography steps has been reduced, the effect of resist residuals and contaminants on the graphene surface also gets reduced resulting in one order higher current density as compared to other patterned CVD graphene current densities reported in^19,20^.

The results obtained has been divided into four main parts

### Effect of Width on the graphene interconnect

The variation of current with width (0.3 to 1.5 µm) of monolayer graphene interconnect is given in Table 1 where the length has been fixed to 1 μm. These widths were particularly studied for the application viewpoint of MQCA based nanoelectronic devices. Since, the nanomagnets used in MQCA based devices are in the range of 135 ×70 ×30 nm^3^ ^39^, hence in order to place the easy axis of the nanomagnets on the graphene interconnect, the minimum width of the graphene interconnect was selected in the range of equal to or more than 300 nm and less than the copper interconnect, so as to maintain the keep-out zone (to have the proper placement of nanomagnets on the interconnect and also to avoid fringing field interactions between nanomagnets).

**Table 1:** Variation of current with the width of Graphene interconnects at room temperature.

Sl. No	Width of the Graphene interconnect in micrometres	Current in microamperes for breakdown voltage-2V and test temperature 300 K
1.	0.3	117.89
2.	0.6	135
3.	1	252.1
4.	1.5	512.4

**Figure 3:**
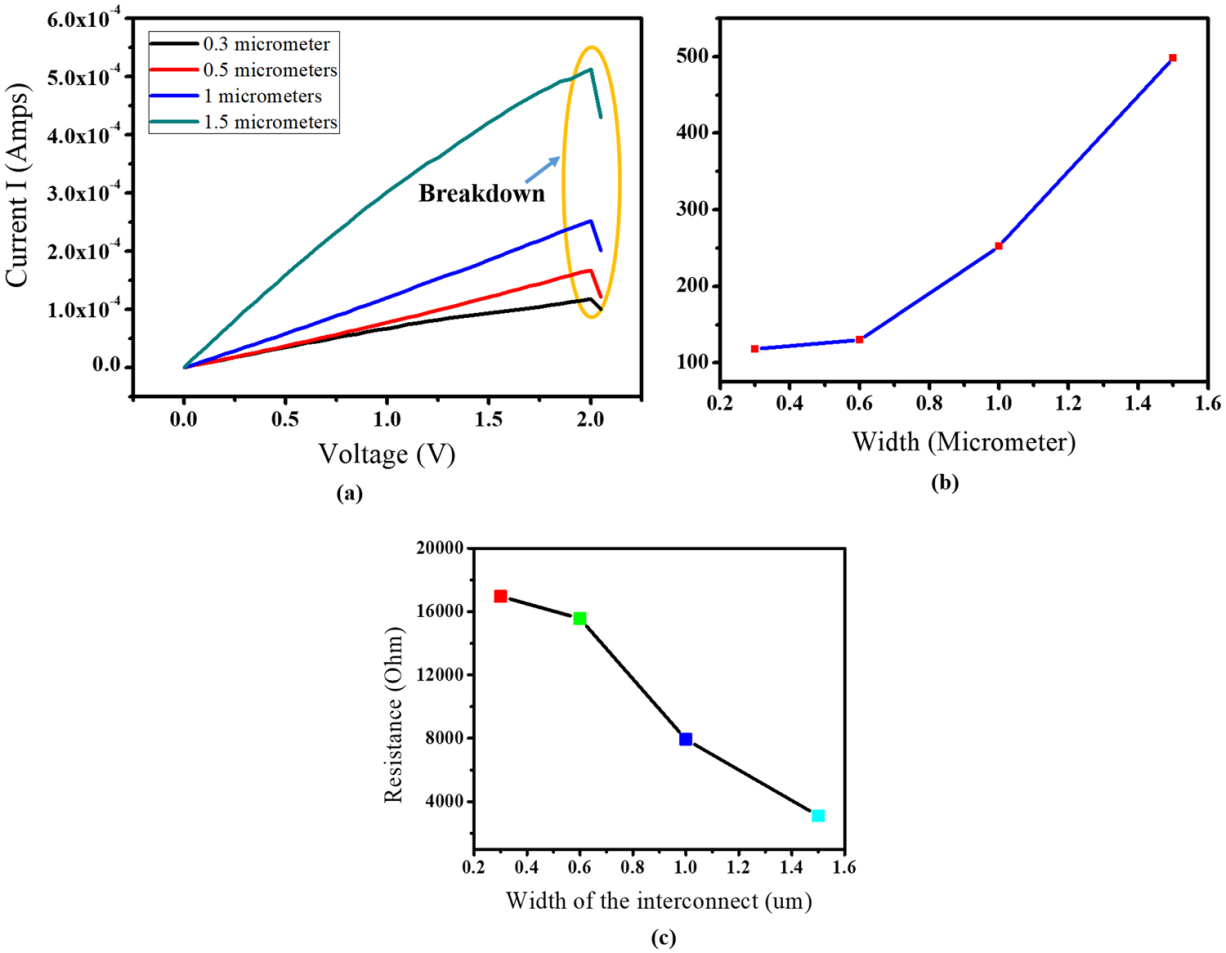
(**a**) Current vs voltage plot up to the electrical breakdown point, (**b**) Width vs current relation and (**c**) Resistance vs Width relation for Monolayer graphene interconnect of different width.

It was observed that with an increase in width of the graphene interconnects, the current also increased. It was found that the current (at breakdown voltage) increases at an increasing rate, though the breakdown current density decreases as shown in Fig. 3(b). It can be observed from Fig. 3(c) that, with the increase in width the resistance decreases due to the availability of higher number of free electrons on the surface of graphene and as a result the current values have increased.

The results obtained indicate that as the width increases the influence of the interconnect boundaries (rough area) decreases leading to lesser scattering and increased conductivity & current^40^.

Breakdown current densities for all the cases were calculated and a maximum current density of 1.18×10^8^ A/cm^2^, was observed which is an order more than state-of-the-art^20^, whereas the minimum was found to be 3.68 ×10^7^ A/cm^2^ for 1.5 μm interconnect at room temperature.

This may be attributed by the reduction in the number of lithography steps as compared to the state of the patterning of CVD Graphene interconnects. Figure 3(a) shows the I-V characteristics of graphene interconnect which are covered by a thin layer of HSQ. It was found that an HSQ coating does not degrade the current carrying capacity of the graphene interconnect that is consistent with previous findings^21^. The voltage and current were found to follow ohm’s law, i.e. *V* α *I*.

Patterning CVD monolayer graphene samples is quite an exhaustive, complicated and expensive process and thus we have formulated a mathematical expression for calculating the current from the width of the interconnects. The relationship between the current and width has been derived from Fig. 3(b) and is found to be typically valid when the graphene interconnects ranges between 300 to 1500 nm and is given as-

1 $$I=a+bw+c{w}^{2}$$

The best fit for the obtained values of the intercept were found to be a = 143.93, b = −177.47 and c = 288.44. ${R}^{2}$ for this fit was found to be 99.91%. I (microampere) and w (micrometer) represent here the current and width of the graphene interconnects and a, b and c are constants respectively. Thus from the above relation, we can calculate the current or width of the interconnects without doing fabrication every time.

### Effect of temperature on the graphene interconnect

**Figure 4:**
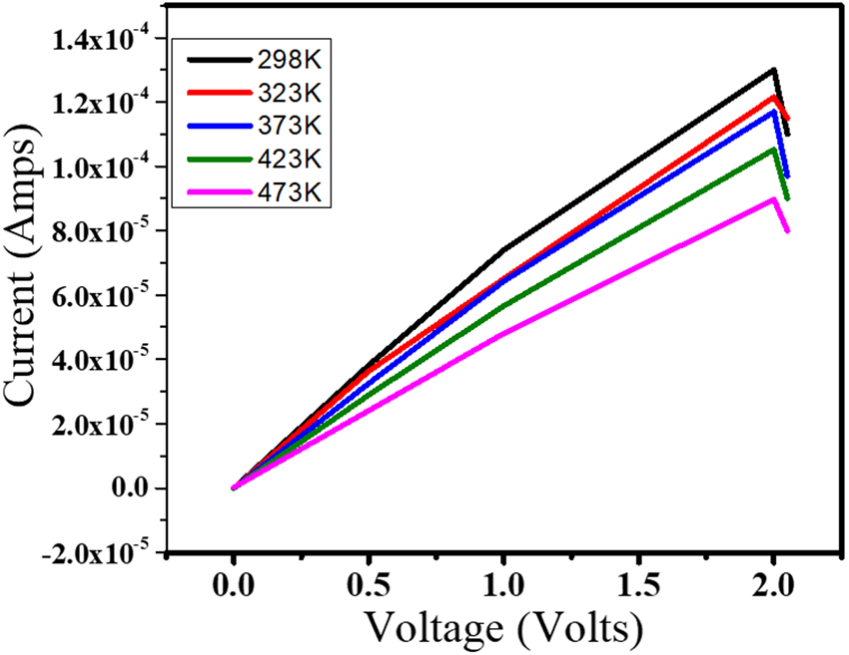
High-temperature current-voltage characteristics of graphene interconnect.

The graphene interconnects between the two metal electrodes on an insulating oxide layer was electrically characterised in the temperature range of T = 298 K to 473 K. The typical I-V characteristics of the CVD monolayer graphene interconnect of a 0.6 µm is given in Fig. 4.

It was observed that with the increase in temperature, the current decreased. The resistivity was found to follow the law *ρ* = *ρ*_0_ + *AT* (A is a positive constant). Therefore, it can be observed from the above figure, that the resistance dependence of temperature can be modelled by the equation.

2 $$R={R}_{0}(1+\alpha (T-{T}_{0}))$$

Where α is the temperature coefficient of resistance. This increase in resistance occurs because of the increase of interaction between charge carriers and the phonons of the graphene nanoribbon.

The sensitivity to temperature was also calculated for this case by using the *Temperature Coefficient of Resistance* (TCR), defined as:

3 $$TCR=\frac{\partial R/R}{\partial T}|T={T}^{\ast }$$

The temperature coefficient of resistance at 298 K for 0.6 µm was found to be 0.00248 K^−1^. Thus it was found that TCR was positive and therefore with increase in temperature, the current decreased.

TCR for all other cases of the study was also calculated and is listed below:-

#### Size effects of TCR

From Table 2, it is evident that as the width of graphene interconnect reduces, the coefficient of α decreases, *i.e*. the sensitivity of the resistivity to temperature variations decreases. The TCR for 1 µm was found to be about 38% larger compared to 300 nm wide interconnects.

**Table 2:** Width of graphene interconnects vs TCR.

Width	α(at K^−1^$$)$$
0.3	0.00168
0.5	0.0019
0.6	0.00248
1	0.0027

**Figure 5:**
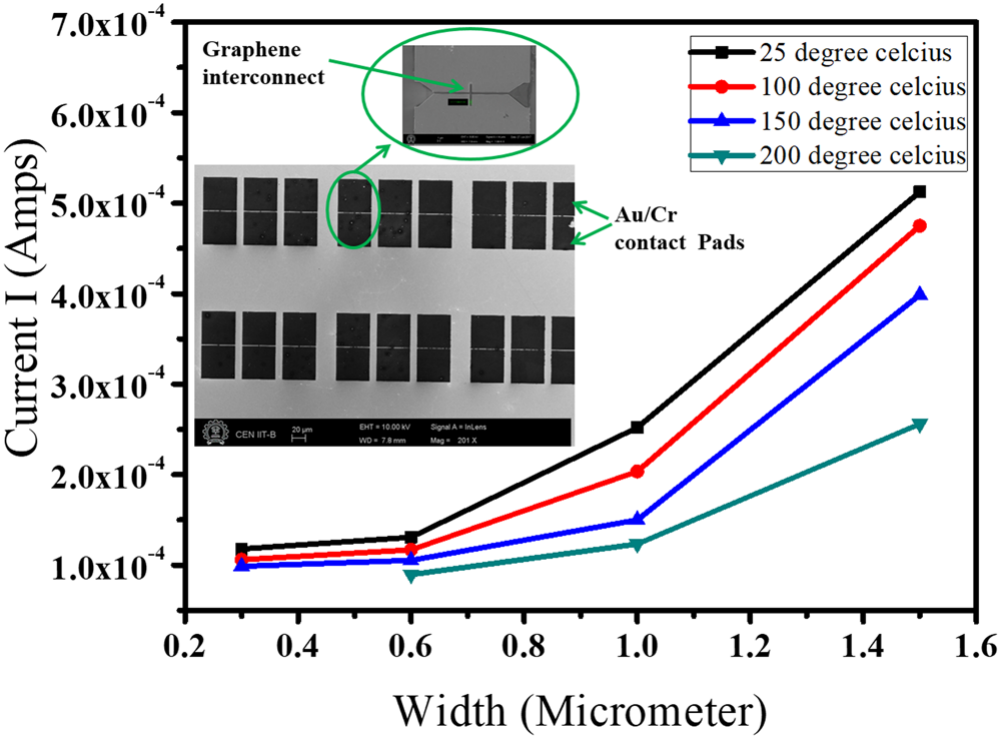
Current vs width plot for monolayer graphene interconnect at different temperatures respectively. The inset shows the SEM image of the patterned graphene interconnects between the metal pads.

The variation of current as a function of a) width of graphene interconnects, and b) temperature ranging from 298 K to 473 K are shown in Fig. 5. The SEM image of the interconnect patterns between the metal pads has been shown in the inset of Fig. 5, where the length of interconnect is fixed in all the cases as l μm and width is varied from 0.3 to 1.5 μm.

We observed that interconnect of width 0.3 μm could not withstand the temperature of 473 K and rather failed after 423 K. The drop in current at 423 K for 0.3 μm width as compared to room temperature was noted to be 17% whereas, for all other cases, the drop in current at 473 K as compared to room temperature was more than 30%. The maximum drop in current was observed for 1.5 μm as 51%. Thus temperature was found to play a significant role in the current-carrying capacity of the graphene interconnect thus leading to decreased current densities.

### Effect of contact resistance on the graphene interconnect

It has been reported in the literature by Withers *et al*.^41^, that Cr/Au forms a good Ohmic contact with graphene and hence the contact resistance (Rc) due to the Cr/Au pads were found to be negligible as compared with the sample resistance. Similarly, as reported by Wei *et al*.^42^ the contact resistance versus contacts inter-spacing plot for CVD grown graphene with Cr/Au contact pads deposited on top of graphene channel was found to be 20 ohms for the contact inter-spacing of 1 µm. The similar observation on the contact resistance was also reported by Cai *et al*.^43^, where Cr/Au metal contact pads were fabricated on Single-layer graphene yielding 33.5 Ω which was found to be compared favourably with the study reported in^42^. We have adopted the procedures followed in the previously reported studies^41–43^, with a similar kind of setup for planning our experiments. The results shown in^41–43^ can also be correlated to the experimentally fabricated monolayer graphene interconnect used in our study.

Moreover from Fig. 3(a), it can also be seen that the I-V curve depicted is a linear curve that follows Ohmic behaviour and thus it can also be inferred from the refs. ^41–43^, the contact resistance is within the range of 20–34 ohm. The total resistance of the proposed fabricated graphene interconnects falls in the range of 3.5–18 KΩ, and thus the impact of the contact resistance (in the order of 20–34 ohm) on the total resistance can be considered as negligible which is also aligned with the findings reported in^17^ by Murali *et al*.

### Electrical breakdown of the graphene interconnects

**Figure 6:**
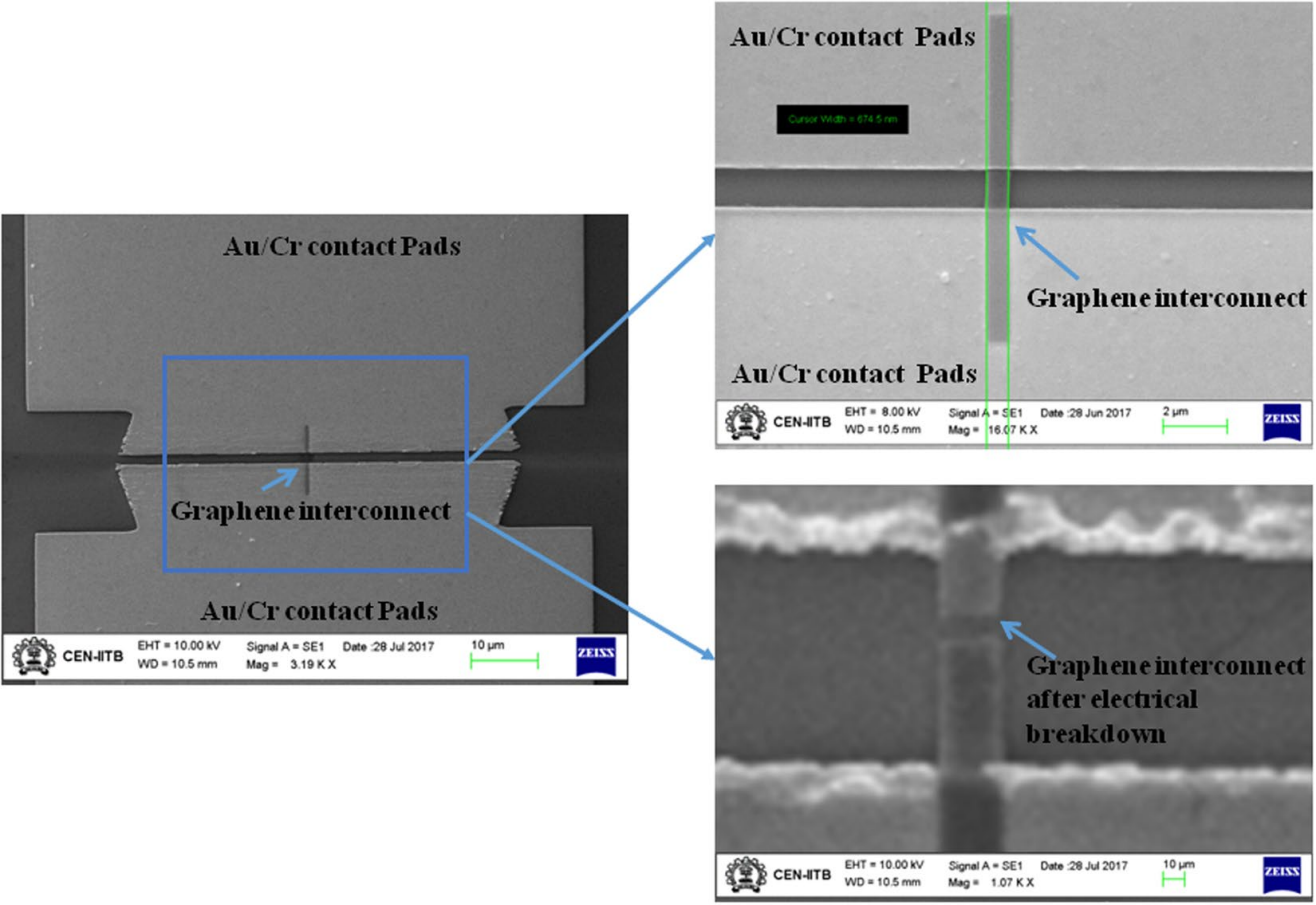
SEM image of (**a**) Monolayer graphene interconnect between metal pads (**b**) at intact condition and (**c**) after electrical breakdown.

Figure 6 shows the SEM images of graphene interconnect covered by HSQ where Fig. 6(a) shows the conducting Graphene interconnect of 600 nm and (b) shows the graphene interconnect after the electrical breakdown. As the voltage was increased, the flow of current produces Joules heating leading to temperature rise across the graphene interconnect. After reaching breakdown voltage of 2 V the graphene interconnect failed at the mid-region, although incipient melting was also observed at the metal pads as heat dissipated equally through them as shown in Fig. 6(c). This may be due to better heat dissipation at the metal pads (Cr/Au) due to higher thermal conductivity of gold-chromium alloy as compared to SiO_2_ substrate on which graphene interconnect is directly lying.

**Figure 7:**
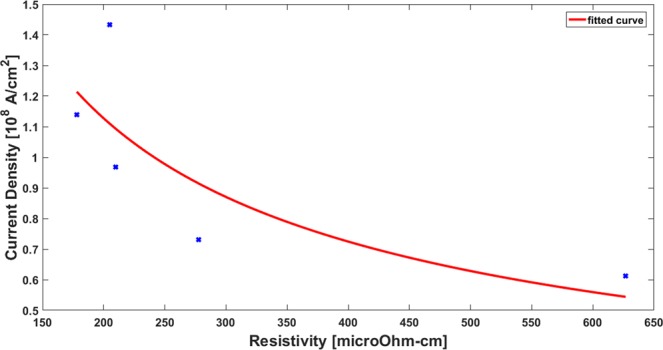
Breakdown current density vs Resistivity plot.

The effect of resistivity on the breakdown current density was analysed on the experimental data using Matlab. From the plot obtained (Fig. 7), it was found that the current density and the resistivity follows a power-law relationship given as-.

4 $${J}_{BR}=C{\rho }^{-b}$$

Where J represents the current density in Ampere/Cm^2^, C and b are constants while $\rho$ represents the resistivity respectively. The best fit was obtained for C = 32.9 ×10^8^ and b = −0.64 with *ρ* having the units of μΩ-cm. ${R}^{2}$ for this fit was found to be 65%.

The exponent term of 0.64 represents the breakdown of the graphene interconnects with increasing resistivity, indicating that higher resistivity is responsible for the degradation of breakdown current density. This higher resistivity can be attributed to the defect morphologies such as the lattice imperfections, in-plane defects and voids, thus resulting in - jJoule’s heating that finally results in the electrical breakdown.

The maximum temperature point of a suspended longitudinal dc biased graphene interconnect was found at the middle point. In steady-state conditions, the distribution of temperature θ(*x*) along a conductor is governed by the heat equation –

5 $$\frac{{d}^{2}\theta (x)}{d{x}^{2}}-\frac{\theta (x)}{{L}_{H}^{2}}=-\frac{q}{k}$$

Where q = ${J}^{2}\rho$, is the volumetric heat generation due to heating, k is the thermal conductivity and ${L}_{H}$ is the thermal heating length defined as

6 $${L}_{H}=\sqrt{\frac{ka}{g}}$$

*g* is the thermal conductance of the GNR, and a is the area of cross-section. Inserting the values of k, g from ref. ^17^, the maximum temperature was found to be 1073 K (800 °C) at x = l/2, where l is the length of the graphene interconnect.

Thus the mechanism of breakdown reported here is believed to be Joule’s heating. Similar phenomenon has been reported by Collins *et al*. in^44^ where, breakdown in Multi-walled carbon nanotube occurred midway between the two electrodes which was precisely due to the dissipative self-heating that produces a peak temperature of 500–700 °C, a range that agrees well with the thermal oxidation of graphite studies as also reported by Yao *et al*. in^45^. Similarly, Murali *et al*. has studied the breakdown event of the graphene nanoribbon as reported in^17^, where the graphene nanoribbon breakdown occurred midway between the two electrodes producing a peak temperature of 700–800 °C. A similar breakdown phenomenon of the graphene interconnect was also observed in our proposed study, where it was found that the graphene interconnects breakdown occurred midway between the two electrodes and that produces a peak temperature of 700–800 °C, inferred from the above calculation that shows that the maximum temperature obtained at x = l/2 was 1073 K (800 °C), where l is the length of the graphene interconnect resulting thermal oxidation of graphite^45^ and maybe intimately connected with self-heating of the graphene interconnects.

Furthermore, in order to explain the constant breakdown voltage with the increment in width, the Power dissipation (Joule’s heating) for all cases was calculated as shown below:-

It can be observed from Table 3 that, the power dissipation increased with the increase in width of the graphene sheet. Also it can be noticed from Fig. 3(b,c), that with the increase of width, the resistance decreases and the current increases.

**Table 3:** Width of graphene interconnects Vs. Power relation.

Sl. No	Width (W) of the sample, µm	I^2^R product value, 10^−4^ W at Breakdown Voltage (2 V)
1.	0.3	2.35
2.	0.6	2.56
3.	1	5.04
4.	1.5	8.24

Thus from the experimental observation, it can be found that:-

7 $${{\rm{I}}}_{1}{{\rm{R}}}_{1}={{\rm{I}}}_{2}{{\rm{R}}}_{2}={{\rm{I}}}_{3}{{\rm{R}}}_{3}={{\rm{I}}}_{4}{{\rm{R}}}_{4}=2{\rm{V}}$$

It can be noted that, although the product of I and R, i.e., breakdown voltage (V) remains the same, for various widths, the quadratic component I play the dominant role in contributing to the self-heating phenomenon which is referred here as the Joule’s heating and the same can be represented using the inequality below and is also tabulated in Table 3.

8 $${{{\rm{I}}}_{1}}^{2}{{\rm{R}}}_{1} < {{{\rm{I}}}_{2}}^{2}{{\rm{R}}}_{2} < {{{\rm{I}}}_{3}}^{2}{{\rm{R}}}_{3} < {{{\rm{I}}}_{4}}^{2}{{\rm{R}}}_{4}$$

Thus, the quadratic component of the current I is not same throughout and therefore the Power dissipation, P = I^2^R is causing the breakdown in the graphene interconnects which is the jJoule’s heating. Also it can be noted that with the wider graphene patterns, both the heat dissipation and heat absorption capability increases due to increase in the surface area. Since the radiative heat transfer is directly proportional to the surface area, increase in Joule’s heating due to the quadratic component of current (I^2^) is therefore compensated by the higher heat dissipation resulting in averaging out the effect of increase in the temperature of the patterned graphene sample and hence the breakdown voltage doesn’t get lowered down even for the wider graphene patterns.

Hence from the experimental observations and the theoretical calculations, the authors intuitively conclude that the mechanism of breakdown is attributed primarily by the self-heating referred to as Joule’s heating.

## Conclusion

The effect of width and temperature on the electrical properties of CVD monolayer graphene has been investigated experimentally. The outcomes of the study are summarised below:

- A simple two-step lithography for patterning CVD monolayer graphene has been used, thus resulting in lower complexity, cost-effective solution and higher current density as compared to the state-of-the-art fabrication methods for CVD graphene.
- The fabricated graphene interconnects were found to have a current density of 1.18 ×10^8^ A/cm^2^ (for 0.3 μm width) that is 100 times (10^6^ A/cm^2^) more than the copper, resulting in outperforming copper-based interconnects.
- Based on our experimental observation, we formulated a generalised equation between the current and width of the CVD monolayer graphene-based interconnect that enables to calculate the current or width values without doing fabrication.
- The current at the breakdown point was found to decrease significantly with the increase in temperature, indicating a positive temperature coefficient of resistance. Further, as width of interconnects increases from 0.3 to 1.5 μm, the current at breakdown voltage was found to increase, at an increasing rate. This is due to decrease in scattering because of lesser contribution of boundaries, as the width of interconnects increases.
- It was observed that the graphene interconnects failed at the mid-segment of the interconnects due to incipient fusion at the breakdown voltage.
- The failure mechanism of the graphene interconnects was analysed. We found that the current density and the resistivity follows a power-law relationship signifying that higher resistivity of the interconnects were also responsible in the degradation of breakdown current density. This higher resistivity can be attributed to the defect morphologies such as the lattice imperfections, in-plane defects and voids, thus resulting in - joule’s heating that finally results in the electrical breakdown.
- Monolayer graphene interconnects on SiO_2_ substrate obeyed Ohm’s law.
- The drop in current at 473 K for nearly all other cases as compared to room temperature was more than 30%.
- The temperature was found to have a profound effect on the graphene interconnects. Results show that while working with lower widths of the monolayer graphene interconnects at high temperatures pose challenges.

Thus with the maximum current density being 1.18 ×10^8^ A/cm^2^ for 0.3 μm graphene interconnect on SiO_2_/Si substrate, i.e. two orders higher than that of conventionally used copper interconnects, Graphene has an enormous potential in outperforming copper wires.
